# Engaging people with lived experience in a national research network: Experience from the Canadian Consortium on Neurodegeneration in Aging

**DOI:** 10.1002/alz.085965

**Published:** 2025-01-09

**Authors:** Jennifer Bethell, Karen Myers, Myrna Norman, Ellen Snowball, Katherine S McGilton

**Affiliations:** ^1^ University of Toronto, Toronto, ON Canada; ^2^ KITE Research Institute, Toronto Rehabilitation Institute ‐ University Health Network, Toronto, ON Canada; ^3^ Engagement of People with Lived Experience of Dementia (EPLED), Canadian Consortium on Neurodegeneration in Aging (CCNA), Montreal, QC Canada

## Abstract

Engagement of People with Lived Experience of Dementia (EPLED) was a new cross‐cutting program introduced as part of the Canadian Consortium on Neurodegeneration in Aging (CCNA) for their second 5‐year phase (from 2019 to 2024). The initiative was supported by the Alzheimer Society of Canada as part of their commitment to the CCNA. EPLED had three objectives: (1) support persons with dementia and care partners to be actively involved in the CCNA research process; (2) work with CCNA research Teams, Cross‐cutting Programs and Partners to develop novel mechanisms and formats to further this collaboration; and, (3) advance the methods of patient engagement in research by embedding evaluation. One of the main activities of EPLED was to recruit and support an Advisory Group of people from across Canada with diverse lived experiences of dementia. Since 2020, EPLED Advisory Group members became involved in various CCNA central and research team activities as well as contributing as co‐presenters and co‐authors in academic and non‐academic venues and as co‐applicants and co‐investigators on research grants. Advisory Group members also took on roles advocating for more people living with dementia in research roles. Key factors described for the success of the Advisory Group were developing trusting relationships, providing education, offering support, being flexible and acknowledging tensions between research, practice and lived experience. Ongoing challenges often related to the gap between research and practice and balancing EPLED’s research objectives with aspirations for advocacy and system‐level change. Looking forward to the third phase of the CCNA, key challenges will include: developing capacity to meet increasing research funding expectations of engagement while also ensuring meaningful engagement, engaging new perspectives by recruiting new Advisory Group members while also retaining expertise from existing members, evaluating the impact of engagement and initiating EPLED‐driven research initiatives.

